# Relationship between Azathioprine metabolites and therapeutic efficacy in Chinese patients with neuromyelitis optica spectrum disorders

**DOI:** 10.1186/s12883-017-0903-5

**Published:** 2017-07-05

**Authors:** Xindi Li, Shenghui Mei, Xiaoqing Gong, Heng Zhou, Li Yang, Anna Zhou, Yonghong Liu, Xingang Li, Zhigang Zhao, Xinghu Zhang

**Affiliations:** 1 Neuroinfection and Neuroimmunology Center, Department of Neurology, Beijing Tiantan Hospital, Capital Medical University, 6 TiantanXili, Dongcheng District, Beijing, 100050 People’s Republic of China; 2 China National Clinical Research Center for Neurological Diseases, Beijing Tiantan Hospital, Capital Medical University, 6 TiantanXili, Dongcheng District, Beijing, 100050 People’s Republic of China; 3 Department of Pharmacy, Beijing Tiantan Hospital, Capital Medical University, 6 TiantanXili, Dongcheng District, Beijing, 100050 People’s Republic of China

**Keywords:** Neuromyelitis optica spectrum disorders (NMOSD), Azathioprine (AZA), Thiopurine S-methyltransferase (TPMT), 6-thioguanine nucleotides (6-TGNs), 6-methylmercaptopurine nucleotides (6-MMPNs)

## Abstract

**Background:**

Neuromyelitis optica spectrum disorders (NMOSD) are demyelinating autoimmune diseases in the central nervous system (CNS) that are characterized by a high relapse rate and the presence of anti-aquaporin 4 antibodies (AQP4-IgG) in the serum. Azathioprine (AZA) is a first-line immunomodulatory drug that is widely used for the treatment of patients with NMOSD. However, the efficacy and safety of AZA vary in different individuals.

**Method:**

Thirty-two patients with NMOSD who regularly took AZA were enrolled in the study at Beijing Tiantan Hospital, Capital Medical University. The efficacy of AZA was evaluated using the expanded disability status scale (EDSS) and the annual relapse rate (ARR). The erythrocyte concentrations of AZA metabolites were detected using an LC-MS/MS method.

**Results:**

The erythrocyte concentrations of 6-thioguanine nucleotides (6-TGNs) and 6-methylmercaptopurine nucleotides (6-MMPNs) were 202.03 ± 63.35 pmol/8*10^8^ RBC and 1618.90 ± 1607.06 pmol/8*10^8^ RBC, respectively. After the patients had received AZA therapy for more than one year, the EDSS score decreased from 5.21 ± 0.24 to 2.57 ± 0.33 (*p* < 0.0001), and the ARR decreased from 1.41 ± 0.23 to 0.36 ± 0.09 (*p* < 0.0001). The 6-TGN and 6-MMPN levels were significantly different between the non-relapsed and relapsed groups (*p* < 0.0001, *p* = 0.006, respectively). A higher ARR was significantly correlated with higher erythrocyte concentrations of 6-TGNs (*p* < 0.0001) and 6-MMPNs (*p* = 0.004).

**Conclusion:**

AZA can reduce the EDSS score and ARR in NMOSD patients. Additionally, the efficacy of AZA is significantly related to the erythrocyte concentrations of 6-TGNs and 6-MMPNs. Within the safe upper limits, a higher concentration of 6-TGNs is associated with better efficacy of AZA.

**Trial registration number:**

ISRCTN16551495, retrospectively registered on May 22, 2017.

## Background

Neuromyelitis optica spectrum disorders (NMOSD) are demyelinating autoimmune diseases in the central nervous system (CNS) that are characterized by the presence of anti-aquaporin four antibodies (AQP4-IgG) in the serum [1]. The clinical disability progressively deteriorates, and the occurrence of relapses increases. Currently, azathioprine (AZA), which is a first-line immunomodulatory drug, is widely used for the prevention of relapse in NMOSD patients [2]. The efficacy of the AZA treatment in NMOSD patients has been mainly evaluated by expanded disability status scale (EDSS) values and the annual relapse rates (ARR). Elsone et al. reported that neurological function improved or remained stable in 78% of patients who received ASA therapy, with a mild reduction in the mean EDSS score from 5.5 to 4 in 103 NMOSD patients [3]. The ARR has been shown to decrease after treatment with AZA [4, 5]. In a Chinese retrospective study, 57.1% of NMOSD patients were in relapse-free status [6]. However, the efficacy and safety of AZA vary in different individuals. Severe adverse reactions to AZA, such as leukopenia and liver dysfunction, may limit its use [7]. Therefore, the identification of indicators to guide the safe and effective use of AZA is very important.

The immunosuppressive effects of AZA are mainly due to its metabolites. AZA rapidly transforms into 6-mercaptopurine (6-MP), which is further converted into thiopurine nucleotides (TPNs), including 6-thioguanine nucleotides (6-TGNs) and 6-methylmercaptopurine nucleotides (6-MMPNs), by a series of competitive enzymes [8]. 6-TGNs are produced by hypoxanthine-guanine phosphoribosyltransferase (HGPRT), and 6-MMPNs, which are the inactive by products of AZA, are formed by thiopurine-methyltransferase (TPMT) [9, 10]. The intracellular accumulation of 6-TGNs and 6-MMPNs can inhibit the biosynthesis of nucleic acids and prevent the proliferation of various lymphocytes [11]. The control of lymphocyte apoptosis is vital to the regulation of the immune system. Therefore, the immunosuppression effect of AZA was correlated with the accumulation of 6-TGNs and 6-MMPNs [12].

The AZA metabolites are mainly mediated by TPMT [13]. Allozymes encoded by certain *TPMT* mutant alleles displayed low TPMT activity that was undetectable after their expression in COS-1 cells [14]. TPMT*2 (238G > C), TPMT*3A (460G > A and719A > G), TPMT*3B (460G > A) and TPMT*3C (719A > G) account for 80–95% of patients with low TPMT activity [15, 16]. Pyrimidine and purine nucleosides, such as 6-TGNs and 6-MMPNs, are transported into the cells by the following two types of human nucleoside transporters: concentrative nucleoside transporters and equilibrative nucleoside transporters. These transporters are encoded by two gene families (i.e., *SLC28* and *SLC29*) [17, 18]. Badagnani et al. reported that CNT3 (i.e., *SLC28A3*) played an important role in the mediation of the cellular entry of a variety of physiological nucleosides and synthetic anticancer nucleoside analog drugs [19]. *SLC28A3* was also reported to have a significant influence on the transport of 6-MP [20]. The relationship between polymorphisms of *SLC28A3* and the erythrocyte concentrations of AZA metabolites in NMOSD has been demonstrated in our former study [21]. Based on the previous study, this study analyzed the relationship between the concentrations of AZA metabolites and the immunosuppressive efficacy of AZA to identify predictive biomarkers for efficacy evaluations of AZA in NMOSD patients.

## Methods

### Subjects

This study analyzed the clinical characteristics, AZA metabolites and genetic polymorphism of prospectively enrolled patients. The study was approved by the Ethics Committee of Beijing Tiantan Hospital Affiliated to Capital Medical University, Beijing, People’s Republic of China (No. KY2015–031-02). Written informed consent was obtained from the patients or from their parents / legal guardians who were under 18 or from the close relatives whose participants had severe disability in the writing hand or the illiteracy. Forty-one patients with NMOSD were enrolled. All patients received steroids during the acute disease stage. The initial dosage of methylprednisolone was 1000 mg for 3 days, which was tapered as follows: 500 mg for 3 days, 250 mg for 3 days and 120 mg for 3 days. Then, oral prednisone (60 mg per day) was administered and slowly withdrawn within 12 weeks. AZA therapy was added at the beginning of the oral prednisone administration. The initial dosage of AZA was 50 mg per day for the first 5 days. If no severe adverse reactions appeared, the dosage of AZA was increased to 100 mg per day. Routine blood tests and hepatic and renal functions were monitored regularly (during the first month of the AZA intake, monitoring was performed once every week; during the 2nd month, monitoring was performed once every two weeks; during the 3rd month and thereafter, monitoring was performed once a month). The treatment was stopped if severe adverse reactions occurred. Nine patients withdrew from the study due to the appearance of severe adverse reactions. If relapse occurred, high-dose steroids were re-introduced and withdrawn as previously described.

The inclusion criteria in this study were as follows:

1. Met the International Consensus Diagnostic Criteria for Neuromyelitis Optica Spectrum Disorder 2015 [1].
2. Onset age: 12 to 80 years.
3. No previous exposure to any immunosuppressive agent.
4. Did not undergo blood transfusion three months before sampling.
5. Received more than 12 months of AZA treatment, and the dose has not been changed within the previous 4 weeks to ensure a stable AZA metabolite profile.

The exclusion criteria were as follows:

1. Intolerance to the AZA treatment due to any severe adverse reaction, such as the leukocyte counts less than 4 × 10^9^/L, other severe cardiovascular disease or hepatopathy.
2. Planned or current pregnancy and/or breast feeding.
3. Other unsuitable characteristics as determined by the clinicians.

### Methods

The disability was measured by the EDSS. The pretherapy EDSS was evaluated at the stable stage (more than one month after relapse), and the post-therapy EDSS was evaluated at the final visit (at least one year after the AZA treatment). The ARR was calculated according to the number of relapses per year. To remove the influence of the pretherapy ARR, we measure the ARR improvement as the pretherapy ARR minus the post-therapy ARR divided by the pretherapy ARR (the value of the ARR improvement was positive). A confirmed relapse was defined as the appearance of new neurological symptoms or worsening of preexisting symptoms that lasted for at least 24 h and was accompanied by an objective neurological change (worsening by 0.5 points on the EDSS or by ≥1.0 points on the pyramidal sign, cerebella, brainstem, or visual functional system scores) in patients who had been neurologically stable or improving in the previous 30 days [6]. The relapsed group included patients who had a confirmed relapse after taking AZA. The non-relapsed group included patients who did not experience any relapse event during the follow-up duration. The AZA efficacy was evaluated based on the changes in the EDSS score and the ARR after the therapy.

CSF and serum samples for the immunological tests, including CSF protein, CSF IgG and AQP4-IgG, were routinely collected during the acute phase before any therapy was administered. Cell-based assays were used for the serum AQP4-IgG detection [22]. Blood samples (5 mL) were collected in vacuum tubes (containing ethylenediaminetetraacetic acid) after 30 days of regular AZA therapy (during the remission phase). After centrifugation at 5000×*g* for 10 min, the plasma was removed, and the white blood cells were stored at −80 °C for genotyping. The erythrocytes were washed twice with 2 ml saline and centrifuged at 10000×*g* for 2 min to remove the supernatant, which was subsequently stored at −80 °C to detect the concentrations of erythrocyte 6-TGNs and 6-MMPNs using our previously reported method (high-performance liquid chromatographic tandem mass spectrometry) [21]. The measurements of TPNs were divided by the body mass index, total daily dose, and mean corpuscular volume to remove the influence of these variables.

### Statistical analysis

SPSS (version 17, SPSS Inc., Chicago, IL, USA) was used for statistical analysis. The chi-square test was used to compare the ARR values (before and after the treatment) and the serum AQP4-IgG concentrations. Nonparametric independent-sample *k-s* test was used to analyze the differences in the EDSS scores. Independent-samples *t-*test was used to analyze the differences in the other related variables. The relationships between the concentrations of the AZA metabolites and ARR improvement were evaluated by 2-tailed *Spearman rank* correlation and *multiple linear-regression,* which was adjusted for onset age, disease duration, therapy duration, pretherapy and post-therapy EDSS scores, and laboratory tests. A two-tailed *p*-value <0.05 was considered statistically significant.

## Results

### Characteristics of NMOSD patients treated with AZA

**Fig. 1:**
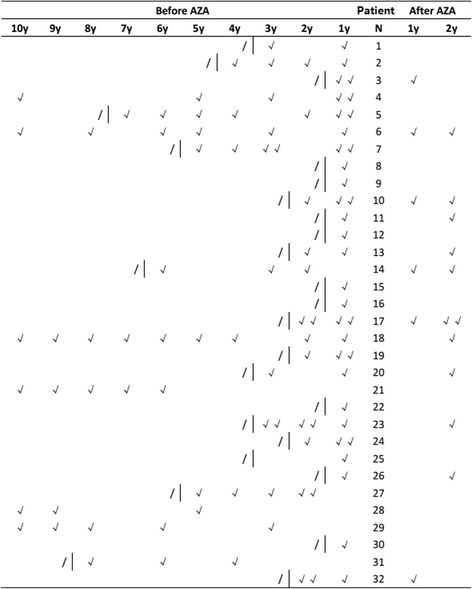
The individual relapse times of each patient within 10 years before AZA therapy and 2 years after AZA therapy. “√” denotes the time of relapse. “I” denotes the beginning of AZA therapy. “/” indicates not available

All 32 NMOSD patients (30 females and 2 males) regularly received AZA therapy. The average onset age was 33.28 years (ranged from 18 to 62, SD = 12.77). The average duration of the disease was 85.56 months (ranged from 19 to 266, SD = 68.18), and the average duration of AZA therapy was 21.50 months (ranged from 12 to 40, SD = 6.19). In total, 21 (65.6%) patients presented with optic neuritis, and 29 (90.6%) patients presented with myelitis. The mean concentration of the CSF protein was 40.41 ± 32.67 mg/dl, and the mean CSF IgG index was 0.53 ± 0.10. Serum anti-AQP4 antibodies were positively detected in 14 (43.75%) patients. The concentrations of erythrocyte 6-TGNs were within the safe upper limits (6-TGN < 450 pmol/8 × 10^8^ RBC), but a few patients had erythrocyte 6-MMPN concentrations above the safe upper limits (5700 pmol/8 × 10^8^ RBC) [23]. The average 6-TGNs concentration was 202.03 pmol/8*10^8^ RBC (ranged from 83.441 to 319.71, SD = 63.35), and the average 6-MMPN concentration was 1618.90 pmol/8*10^8^ RBC (ranged from 235.04 to 7498.89, SD = 1607.06). The mean EDSS score decreased from 5.21 ± 0.24 to 2.57 ± 0.33 (*p* < 0.0001), and the mean ARR decreased from 1.41 ± 0.23 to 0.36 ± 0.09 (*p* < 0.0001). Detailed information is provided in Table 1. The individual relapse events within 10 years before AZA therapy and 2 years after AZA therapy are shown in Fig. 1, and two patients (No. 6 and No. 14) had worse ARRs after AZA therapy.

**Table 1:** The general characteristics of NMOSD patients

Variable	Patients (*n* = 32)
Mean Age of onset, year	33.28 (15–62)
Sex (n%)	
Female	30 (93.75%)
Male	2 (6.25%)
Mean BMI	24.17 (17.57–31.65)
Mean Disease duration, months	85.56 (19–266)
Mean Duration of AZA therapy, months	21.50 (12–40)
Clinical symptoms at onset (n%)	
Optic neuritis	21 (65.6%)
Bilateral Optic neuritis	17 (53.13%)
Myelitis	29 (90.6%)
Optic neuritis and Myelitis	19 (59.3%)
Mean Pre-therapy EDSS score	5.21 ± 0.24 (2.5–8)
Mean Post- therapy EDSS score	2.57 ± 0.33 (0–7)
Mean Concentration of CSF protein, mg/dl	40.41 ± 32.67 (12–184)
Mean Concentration of CSF IgG, mg/ml	0.04 ± 0.03 (0.01–0.19)
CSF IgG index (n%)	0.53 ± 0.10 (0.39–0.83)
Serum anti-AQP4 antibodies (n%)	14 (43.75%)
Mean Concentration of 6-TGNs, pmol/8 × 10^8^ RBC	202.03
Mean Concentration of MMPNs, pmol/8 × 10^8^ RBC	1618.90
Relapse, n%	12/32 (37.5%)
Pre- therapy ARR	1.42 ± 0.23 (0.2–3.5)
Post- therapy ARR	0.36 ± 0.09 (0–1.72)

*NMOSD* Neuromyelitisoptica spectrum disorders, *AZA* Azathioprine, *AQP4* anti-aquaporin 4, *BMI* Body mass index, *ARR* Annual relapse rate, *EDSS* Expanded disability status scale, *CSF* cerebrospinal fluid, *IgG* Immunoglobulin G

### The differences in the related variables between the non-relapsed and relapsed groups

Except for the EDSS score, other variables were normally distributed with a homogeneity of variance. The mean values of the normalized 6-TGN levels (1.12 vs. 0.70, *p* < 0.0001) and 6-MMPN levels (10.60 vs. 3.62, *p* = 0.006) were significantly different between the non-relapsed and relapsed groups (See Table 2). No significant differences were observed in the other variables.

**Table 2:** The difference of related variables between non-relapsed and relapsed groups

Variable	Non-relapsed (*n* = 20)	Relapsed (*n* = 12)	Mean Difference	Std. Error Difference	*p*-value
Age of onset, year	33.35	33.16	−0.183	4.740	0.969
Mean BMI	23.89	24.63	0.741	1.415	0.604
Disease duration, months	80.40	94.17	13.77	25.81	0.589
Duration of AZA therapy, months	22.10	20.50	−1.6	2.278	0.488
Mean CSF protein, mg/dl	39.82	41.43	1.613	13.275	0.904
Mean Concentration of CSF IgG, mg/ml	0.040	0.032	−0.008	0.014	0.577
CSF IgG index (n%)	0.546	0.494	−0.519	0.039	0.199
Serum anti-AQP4 antibodies (n%)	10 (50%)	4 (33.3%)	/	/	0.471
Pre-therapy EDSS score	5.17	5.29	/	/	0.687
Post- therapy EDSS score	2.43	2.83	/	/	0.893
Normalized concentration of 6-TGNs	1.12	0.70	−0.418	0.09	<0.0001*
Normalized concentration of 6-MMPNs	10.60	3.62	−6.983	2.32	0.006*

NMOSD, *AZA* Azathioprine, *BMI* Body mass index, *ARR* Annual relapse rate, *AQP4* anti-aquaporin 4, *EDSS* Expanded disability status scale, *6-TGN* 6-thioguanine nucleotides, *6-MMPN* 6-methylmercaptopurine nucleotides, *CSF* cerebrospinal fluid, *IgG* Immunoglobulin G. All two-tailed *P*-value <0.05 was considered statistically significant

### Relationships between the post-therapy ARR and the erythrocyte concentrations of 6-TGNs and 6-MMPNs

**Fig. 2:**
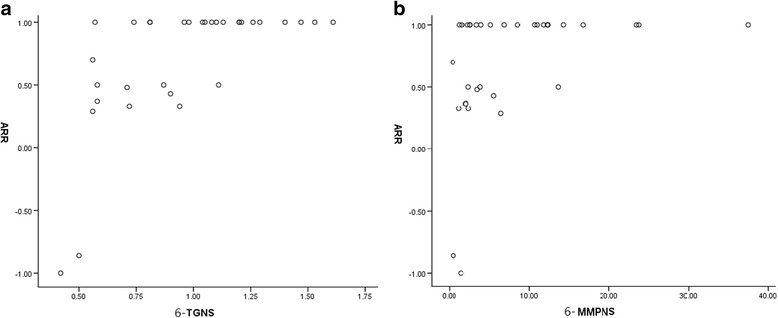
**a** Plot of ARR improvements vs. 6-TGNs. **b** Plot of ARR improvements vs. 6-MMPNs

The normalized erythrocyte concentrations of 6-TGNs and 6-MMPNs followed a normal distribution. However, ARR improvement did not follow a normal distribution. Higher ARR improvements were significantly correlated with higher erythrocyte concentrations of both 6-TGNs (correlation coefficient (R) = 0.679, *p* < 0.0001) and 6-MMPNs (*R* = 0.493, *p* = 0.004) (See Table 3 and Fig. 2).

**Table 3:** The correlation between ARR improvements and normalized erythrocyte concentrations of AZA metabolites

Variable	R	*P*-value
Normalized Concentration of 6-TGNs	0.679	<0.0001*
Normalized Concentration of MMPNs	0.493	0.004*

*6-TGN* 6-thioguanine nucleotides, *6-MMPN* 6-methylmercaptopurine nucleotides, *R* Spearman’s correlation coefficient. All two-tailed *P*-value <0.05 was considered statistically significant

The ARR improvement (in log) was normally distributed. According to the multiple linear-regression, the ARR improvement (in log) was significantly influenced by the erythrocyte concentrations of 6-TGNs (adjusted R square = 0.206, constant = 0.417, standardized coefficients beta = −0.489, *p* = 0.013). The remaining variables, including the erythrocyte concentration of 6-MMPNs, onset age, disease duration, treatment duration, pretherapy and post-therapy EDSS scores, mean CSF protein, mean concentration of CSF IgG and the CSF IgG index, were excluded from the analysis due to underpowered statistical significance (*p* > 0.05) (See Table 4).

**Table 4:** The linear- regression between multiple variables and ARR improvements (in log)

Variable	Standardized coefficients- Beta	T	*P*-value
Constant (SE)	0.417 (0.116)	3.580	0.002
Normalized Concentration of 6-TGNs	−0.489	−2.689	0.013*
Excluded variables			
Age of onset, year	0.156	0.853	0.403
Disease duration, months	0.094	0.509	0.616
Treatment duration, months	−0.200	−1.106	0.281
Pre-therapy EDSS score	0.034	0.176	0.862
Post-therapy EDSS score	0.050	0.266	0.793
Mean CSF protein, mg/dl	−0.038	−0.198	0.845
Mean Concentration of CSF IgG, mg/ml	−0.158	−0.855	0.402
CSF IgG index (n%)	−0.287	−1.636	0.116
Normalized Concentration of MMPNs	−0.150	−0.751	0.461

*NMOSD*, *AZA* Azathioprine, *BMI* Body mass index, *ARR* Annual relapse rate, *EDSS* Expanded disability status scale, *6-TGN* 6-thioguanine nucleotides, *6-MMPN* 6-methylmercaptopurine nucleotides. All *P*-value <0.05 was considered statistically significant

## Discussion

The AZA treatment had a good efficacy in the NMOSD patients, and dramatic reductions in the EDSS scores (from 5.21 to 2.57, *p* < 0.0001) and ARR values (from 1.41 to 0.36, *p* < 0.0001) were observed. These results may be partially due to the concomitant use of corticosteroids, which was reported in the literature [6, 24]. The reduced ARR in our study was consistent with two large cohort studies in which the ARR decreased from 2.20 to 0.89 (*p* < 0.0001) and from 1.5 to 0 (*p* < 0.00005) [3, 5].

Elsone et al. posed an interesting question of whether the apparent reduction in relapses was simply a ‘regression to the mean,’ reflecting the natural course of the illness, or an immune-suppression effect of the AZA treatment [3]. In our study, no significant differences were observed in the pretherapy EDSS scores or onset of immunological status, including the CSF protein, CSF IgG index and serum AQP4-IgGs, between the relapsed and non-relapsed groups, and none of above parameters were correlated with the ARR improvement, which indicated homogeneity in individual disease activities. Therefore, we explored the relationships between the concentrations of the AZA metabolites and the post-therapy ARR. The mean normalized 6-TGNs levels (1.12 vs. 0.70, *p* < 0.0001) and mean normalized 6-MMPNs levels (10.60 vs. 3.62, *p* = 0.006) were significantly higher in the non-relapsed patients than in the relapsed patients. A greater ARR improvement was correlated with higher erythrocyte concentrations of 6-TGNs (*R* = 0.679, *p* < 0.0001) and 6-MMPNs (*R* = 0.493, *p* = 0.004). 6-TGNs are the main immunosuppressive compounds and have been proposed to induce the activation of non-specific apoptotic pathways in proliferating lymphocytes by distorting DNA and weakening its repair system, which leads to apoptosis [25]. 6-MMPNs, which are strong inhibitors of purine de novo synthesis (PDNS), can block the proliferation of various types of lymphocyte lines [26]. In addition, the AZA metabolites can also induce a very specific apoptosis pathway in the CD4+ subset of CD28 co-stimulated T lymphocytes [12]. NMOSD is a CNS immunological disease that can be mediated by various lymphocytes, including Th17 lymphocytes, B lymphocytes and plasma-blasts [27]. After inhibiting the proliferation of lymphocytes by high levels of 6-TGNs and 6-MMPNs, the activation of immunoreactions decreased, and the mean ARR of the NMOSD patients subsequently declined.

The immunosuppressive effect of AZA was hypothesized to be achieved via a balanced contribution of pro-apoptotic (6-TGNs) and anti-metabolic (methylated ribonucleotides) pathways [8]. However, we found that the ARR improvement was only significantly influenced by the erythrocyte concentration of 6-TGNs (standardized coefficients beta = −0.489, *p* = 0.013), which could be due to the pro-apoptosis pathways in which lymphocytes are induced by 6-TGNs, causing widespread damage to lymphocytes in the short-term treatment, and the lethality of the anti-metabolic effect increased with the gradual accumulation of 6-TGNs levels in the long-term treatment [8]. Therefore, we believe that 6-TGNs may play a more important role in the immunosuppressive efficacy than previously thought, particularly during the early remission phase of NMOSD.

Furthermore, the internal variables that influence the erythrocyte concentrations of the AZA metabolites are unknown. The genetic polymorphisms of TPMT [28, 29] can affect TPMT activity, which directly regulates the formation of 6-MMPNs and subsequently influences the 6-TGNs concentration. However, in some recently published articles, no correlation among TPMT activity, 6-TGNs levels and 6-MMPNs levels was observed [30]. We found that rs10868138 (*SLC28A3*) was associated with a higher erythrocyte concentration of 6-TGNs, and rs12378361 (*SLC28A3*) was associated with a lower erythrocyte concentration of 6-TGNs in our former study [21]. The *SLC28A3* gene families correspond to human concentrative nucleoside transporters 3 (CNT3), which appears to be the best drug transporter because it can efficiently transport most of the pyrimidine and purine nucleoside analogs [31]. Our results indicated that the nucleoside transporter encoded by *SLC28A3* (rs10868138) may participate in the metabolism of AZA by transporting 6-TGNs out of the cells, and *SLC28A3* (rs12378361) may help transport 6-TGNs into the cells. In addition to its expression in the membranes of erythrocytes, CNT3 is also present in primary lymphocytes [32], monocytes, monocyte-derived macrophages, and monocyte-derived dendritic cell membranes [33]. Therefore, genetic polymorphisms of *SLC28A3* (encoding CNT3) may influence the activity of NMOSD by altering the concentration of intracellular 6-TGNs in various immune cells. However, considering that *SLC28A3* is not the only factor that influences the concentration of intracellular 6-TGNs, the direct correlation between the polymorphisms of *SLC28A3* and the outcomes of NMOSD must be investigation in further studies.

The disease activity in NMOSD is highly variable among individual patients, and some patients experience AZA efficacy after 6 months to 18 months in clinical practice. Some limitations were present in this study, including the use of patients from a single center, the small sample size and the short follow-up durations. Our preliminary conclusions must be verified in further large cohort studies with long follow-up durations.

## Conclusion

AZA treatment in NMOSD patients can dramatically reduce post-therapy EDSS scores and ARRs, and this effect is significantly correlated with the erythrocyte concentrations of AZA metabolites, particularly 6-TGNs. A higher erythrocyte concentration of 6-TGNs is associated with better efficacy of AZA below the safe upper limits.

## Abbreviations

6-MMPNs: 6-methylmercaptopurine nucleotides
6-MP: 6-mercaptopurine
6-TG: 6-thioguanine
6-TGNs: 6-thioguanine nucleotides
ALL: Acute lymphoblastic leukemia
AQP4-IgG: Anti-aquaporin 4 antibodies
ARR: Annual relapse rates
AZA: Azathioprine
CNS: Central nervous system
EDSS: Expanded disability status scale
HGPRT: Hypoxanthine-guanine phosphoribosyltransferase
HWE: Hardy-Weinberg equilibrium
MAF: Minor allele frequency
NMOSD: Neuromyelitis optica spectrum disorders
PDNS: Purine de novo synthesis
SD: Standard deviation
SLC28A: Solute carrier family 28
SLC29A: Solute carrier family 29
SNPs: Single nucleotide polymorphisms
TPMT: Thiopurine S-methyltransferase
TPNs: Thiopurine nucleotides
